# Coordination and Crystallization Molecules: Their Interactions Affecting the Dimensionality of Metalloporphyrinic SCFs

**DOI:** 10.3390/molecules20046683

**Published:** 2015-04-15

**Authors:** Arkaitz Fidalgo-Marijuan, Eder Amayuelas, Gotzone Barandika, Begoña Bazán, Miren Karmele Urtiaga, María Isabel Arriortua

**Affiliations:** 1 Departamento de Mineralogía y Petrología, Facultad de Ciencia y Tecnología, Universidad del País Vasco (UPV/EHU), Apdo. 644, 48080 Bilbao, Spain; E-Mails: arkaitz.fidalgo@ehu.eus (A.F.-M.); eder.amayuelas@ehu.eus (E.A.); bego.bazan@ehu.eus (B.B.); karmele.urtiaga@ehu.eus (M.K.U.); maribel.arriortua@ehu.eus (M.I.A.); 2 Departamento de Química Inorgánica, Facultad de Ciencia y Tecnología, Universidad del País Vasco (UPV/EHU), Apdo. 644, 48080 Bilbao, Spain; 3 BCMaterials, Parque Tecnológico de Zamudio, Ibaizabal Bidea, Edificio 500-Planta 1, 48160 Derio, Spain

**Keywords:** metalloporphyrin, Solid Coordination Frameworks (SCFs), crystal structure, dimensionality

## Abstract

Synthetic metalloporphyrin complexes are often used as analogues of natural systems, and they can be used for the preparation of new Solid Coordination Frameworks (SCFs). In this work, a series of six metalloporphyrinic compounds constructed from different *meso* substituted metalloporphyrins (phenyl, carboxyphenyl and sulfonatophenyl) have been structurally characterized by means of single crystal X-ray diffraction, IR spectroscopy and elemental analysis. The compounds were classified considering the dimensionality of the crystal array, referred just to coordination bonds, into 0D, 1D and 2D compounds. This way, the structural features and relationships of those crystal structures were analyzed, in order to extract conclusions not only about the dimensionality of the networks but also about possible applications of the as-obtained compounds, focusing the interest on the interactions of coordination and crystallization molecules. These interactions provide the coordination bonds and the cohesion forces which produce SCFs with different dimensionalities.

## 1. Introduction

The challenges of the development model of modern societies move us toward technological advances in many fields. The design and development of new materials allow us to open new opportunities both to energy and material levels. Among new materials, the Solid Coordination Frameworks (SCFs) are a class of multifunctional materials with a wide range of applications in various fields, such as gas storage and separation [1,2], nonlinear optics [3,4], ferroelectricity [5], conductivity [6], magnetism [7], luminescence [8], biomedical imaging [9], chemical sensing [10], drug delivery [11] and heterogeneous catalysis [12,13] among others.

The functions and properties of the molecules forming the crystal structure of these new materials are of great importance on the material behavior. In this sense, metalloporphyrins are complexes that are being used in the generation of some of such materials because they belong to a class of multifunctional biomolecules that play a central role in natural processes in which the transfer of photons, electrons, ions and molecules occurs. Thus, metalloporphyrins-based systems are inspired on these biomolecules to mimic their natural functions and move to both molecular level structured systems and nanotechnological devices [14,15,16,17,18,19,20,21,22].

**Scheme 1:**
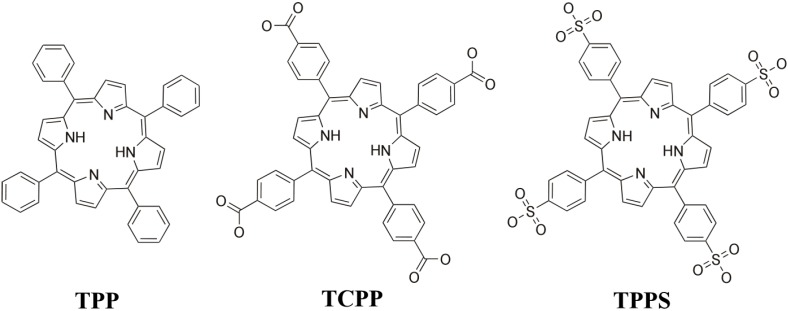
Lewis structure for the selected porphyrins.

In recent years, our research group has been working on the synthesis and characterization of metalloporphyrin-based SCFs [23,24,25,26]. Among the wide variety of available porphyrins, we have chosen a series of *meso*-substituted porphyrins (Scheme 1), such as the *meso*-tetra(4-phenyl)porphyrin (TPP), the *meso*-tetra(4-carboxyphenyl)porphyrin (TCPP), and the *meso*-tetraphenyl porphine-tetrasulfonate (TPPS) combined with iron, cobalt and copper bio-ions.

In this way, using solvothermal, microwave and slow evaporation synthesis techniques, we have obtained six different compounds exhibiting dimensionalities from 0D to 2D. In this work we report on two 0D compounds (a monomer [CuTCPP]·6DMF (**1**) and a dimer μ-O-[FeTCPP]_2_·16DMF (**2**)), three 1D compounds, (([FeTPPbipy]^•^)_n_ (**3**), [CoTPP(bipy)]·([CoTPP])_0.22_·(TPP)_0.78_ (**4**) and [CoTPPS_0.5_(bipy)(H_2_O)_2_]·6H_2_O (**5**)), and a 2D compound ([FeTCPP] (**6**)). The objective of this work is to study the structural features and relationships of those six compounds in order to extract conclusions not only about the dimensionality of the networks but also about possible applications of the as-obtained compounds. With this aim, they have been structurally characterized by single crystal X-ray diffraction, IR spectroscopy and elemental analysis.

## 2. Results and Discussion

**Figure 1:**
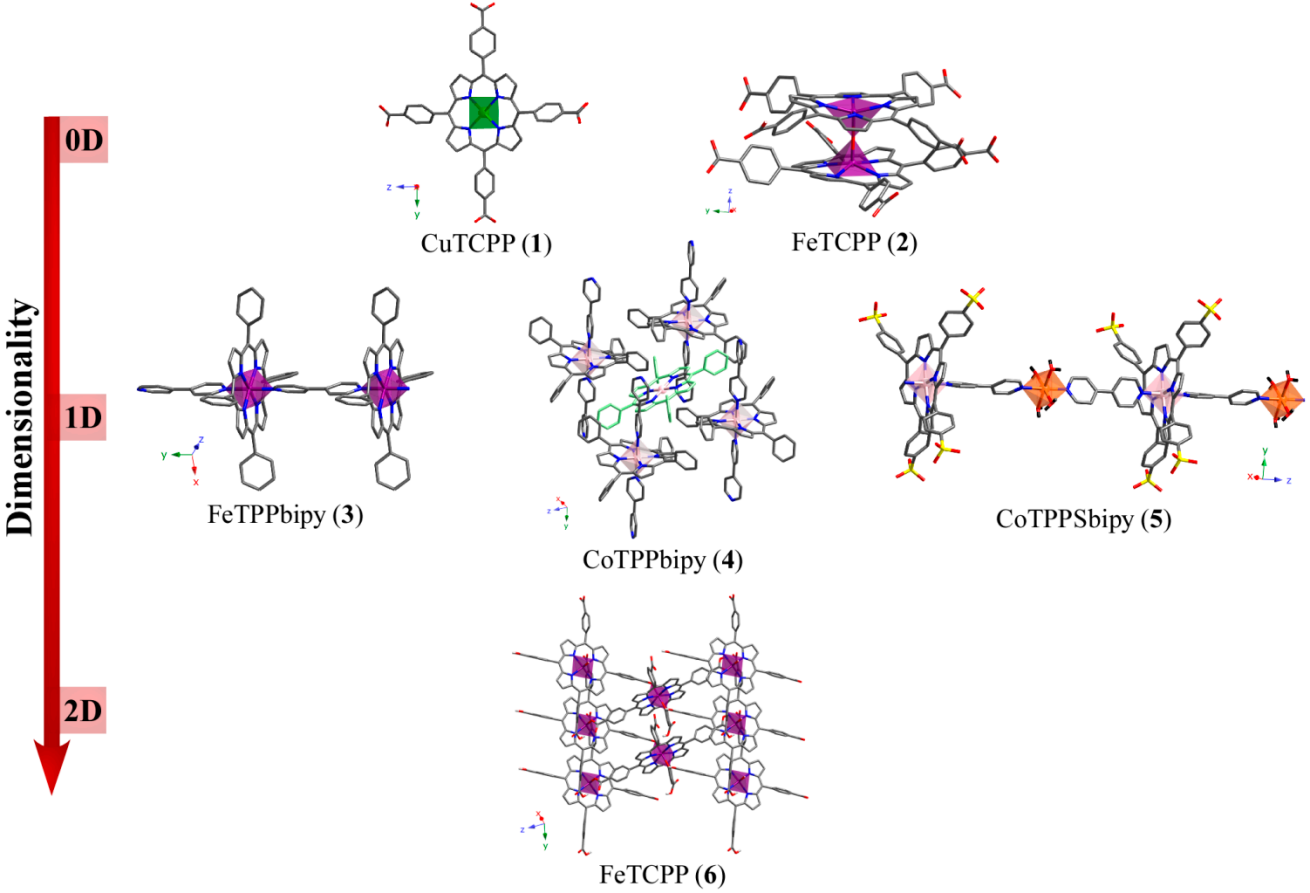
Dimensionality-based classification for compounds **1**– **6**.

As shown in Figure 1, taking into account the dimensionality of the covalent array, compounds **1** to **6** were classified in 0D, 1D and 2D. Table 1 collects the crystal data and structure refinement parameters for compounds **1** to **6**.

**Table 1:** Crystal data and structure refinement for compounds **1**– **6**.

Compound	1	2	3	4	5	6
structural formula	[CuTCPP]·6DMF	μ-O-[FeTCPP]_2_·16DMF	([FeTPPbipy]^•^)_n_	[CoTPP(bipy)]·([CoTPP])_0.22_·(TPP)_0.78_	[CoTPPS_0.5_(bipy) (H_2_O)_2_]·6H_2_O	[FeTCPP]
empirical formula	C_66_H_68_CuN_10_O_14_	C_144_H_168_Fe_2_N_24_O_33_	C_54_H_36_FeN_6_	C_98_H_65.56_Co_1.22_N_10_	C_32_H_36_CoN_4_O_14_S_2_	C_48_H_27_FeN_4_O_8_
*F*_w_, g·mol^−1^	1288.85	2874.71	824.74	1455.06	811.62	843.59
cryst system	Monoclinic	Monoclinic	Monoclinic	Monoclinic	Tetragonal	Monoclinic
space group	*C2/c*	*C*2/c	*C*2/c	*C*2/*c*	*I* 4_1_/*a*	*P*2_1_
*a*, Å	34.4312(9)	39.3340(4)	21.6833(8)	25.1252(4)	17.9776(2)	11.0195(2)
*b*, Å	22.2237(6)	19.8329(2)	11.0827(4)	11.7811(2)		8.8470(2)
*c*, Å	8.3687(2)	16.0292(2)	17.6206(6)	23.9790(4)	22.3567(3)	20.0191(4)
α, deg						
β, deg	103.783(2)	98.4180(10)	97.354(3)	93.5960(10)		102.902(2)
γ, deg						
*V*, Å ^3^	6219.2(3)	12369.8(2)	4199.6(3)	7083.9(2)	7225.55(15)	1902.38(7)
*Z*	4	4	4	4	8	2
ρ*_obs_*, ρ*_cal_*, g·cm^−3^	1.346(5), 1.376	1.575(5), 0.911	1.309(5), 1.304	1.371(6), 1.364	1.488(4), 1.492	1.478(4), 1.473
Crystal size, mm	0.87 × 0.07 × 0.03	0.21 × 0.12 × 0.05	0.34 × 0.07 × 0.07	0.26 × 0.19 × 0.06	0.12 × 0.12 × 0.02	0.36 × 0.15 × 0.01
μ, mm^−1^	1.121	2.304	0.405	2.761	5.445	3.728
absorption correction	Analytical	Analytical	Analytical	Multi-scan	Analytical	Numerical
radiation, λ, Å	1.54184	1.54184	0.71073	1.54184	1.54184	1.54184
temperature, K	100.0(2)	100.0(2)	100.0(2)	100.0(2)	100.0(2)	100.0(2)
reflns collected, unique	25703, 4660 (*R*_int_ = 0.067)	50744, 12049 (*R*_int_ = 0.04)	10334, 3907 (*R_int_* = 0.04)	25120, 7352 (*R_int_* = 0.0405)	23662, 3774 (*R_int_* = 0.096)	20868, 6320 (*R_int_* = 0.048)
final *R* indices [I > 2σ(I)]	R1 = 0.0481, wR2 = 0.063	R1 = 0.0608, wR2 = 0.1867	R1 = 0.0351, wR2 = 0.0714	R1 = 0.0416, wR2 = 0.1095	R1 = 0.0669, wR2 = 0.1845	R1 = 0.0378, wR2 = 0.0877
*R* indices (all data)	R1 = 0.136, wR2 = 0.1513	R1 = 0.0788, wR2 = 0.2025	R1 = 0.0513, wR2 = 0.0738	R1 = 0.0438, wR2 = 0.1115	R1 = 0.0856, wR2 = 0.2040	R1 = 0.0475, wR2 = 0.0921
GOF on *F^2^*	0.956	1.063	0.909	1.056	1.051	1.026
parameters/restraints	421/0	555/0	279/0	504/0	274/3	554/1

### 2.1. 0D Crystal Structures

**Figure 2:**
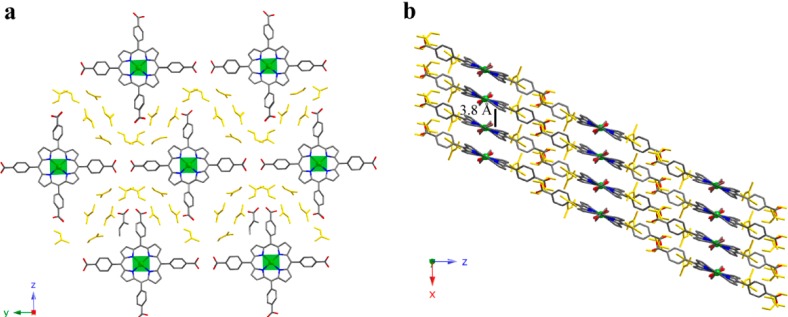
(**a**) View of the H-bonded 2D supramolecular layer and (**b**) packing for compound **1**. (Cu: green, C: grey, N: dark blue, O: red, crystallization DMF molecules: yellow). H atoms have been omitted for clarity.

The crystal structure of compound **1** shows CuTCPP monomeric units. The copper atom is in a four-coordinated square planar environment being the Cu-N distances in the narrow range of 1.998(2) to 2.003(2) Å. These coordination entities crystallize as shown in Figure 2a, where each porphyrinic unit is surrounded by another six, producing an H-bonded 2D layer on the *yz* plane. The robust intralayer H-bonding system involving the DMF solvent molecules located between porphyrins molecules maintains the stability of the supramolecular layers. Those layers are stacked along the [100] direction (Figure 2b) sustained by interporphyrin π-π interactions (3.8 Å) and hydrogen bonds among the monomers and DMF molecules of each layer.

**Figure 3:**
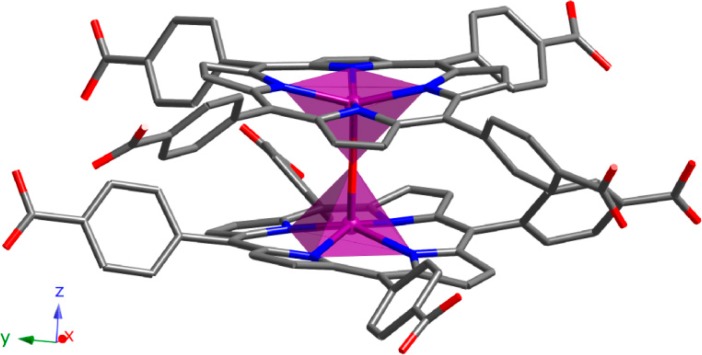
Dimeric unit for compound **2**. (Fe: purple, C: grey, N: dark blue, O: red). H atoms have been omitted for clarity.

Compound **2** is another example of a 0D structure, but in this case it exhibits dimeric units. As observed in Figure 3, two FeTCPP rings are bonded by a bridging O atom. The iron atom is in a five-coordinated square pyramidal environment, displaced by 0.445 Å from the mean porphyrin plane towards the oxo-bridge, and forms a nearly linear Fe-O-Fe angle (179.78°). The Fe-N distances are in the narrow range of 2.077(2) to 2.087(2) Å, while the Fe-O distance is 1.7597(4) Å. These distances and angles are typical for high-spin iron(III) μ-oxo dimers [27]. The macrocyclic rings are essentially parallel to each other, the angle between the two central N_4_ planes being 0.31°. The relative orientations of the two porphyrin rings make an average N-Fe-Fe'-N' dihedral angle (torsion angle of 33.62°) to accommodate the peripheral carboxylic groups. 

**Figure 4:**
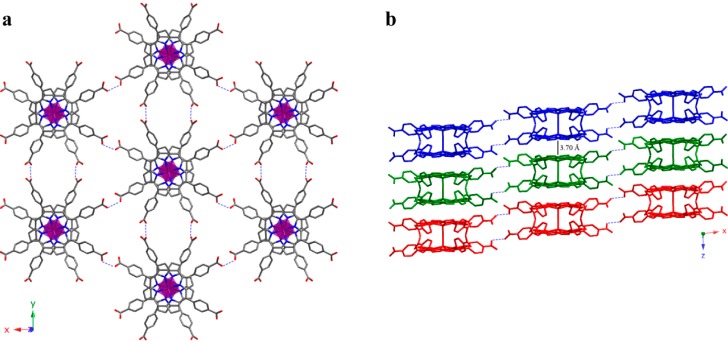
(**a**) View of the H-bonded 2D layer for compound **2**; (**b**) Stacking of the 2D layers for compound **2**, where each layer is shown in a different colour. Intralayer H-bonds are shown as dashed lines. (Fe: purple, C: grey, N: dark blue, O: red). H atoms have been omitted for clarity.

These coordination entities crystallize as shown in Figure 4a. Each dimer is surrounded by another six, producing an H-bonded 2D supramolecular layer on the *xy* plane. The robust intralayer H-bonding system is generated between adjacent porphyrins carboxylate groups maintaining the stability of the layers.

The H-bonded 2D layers are stacked along the [001] direction, sustained by π-π interactions (3.5–3.9 Å) among the dimers of each layer (Figure 4b). Crystallization DMF molecules are located in the voids generated between dimers, unfortunately, these molecules have not been located in the structure refinement process. The resulting solvent accessible volume, removing the DMF molecules, was analyzed with the PLATON program [28], showing a potential solvent volume of 5836.2 Å^3^ (47.2% of the unit cell) and a calculated effective volume of 2470.1 Å^3^. Taking into account the single crystal experimental density, the initial weight loss observed in the thermogravimetric analysis and the calculated free effective volume, we have estimated the presence of 16 DMF molecules per formula unit [26]. These DMF molecules should be located on the two types of voids in the crystal structure (along the c axis and interweaving the 2D layers). Catalytic activity tests towards oxidation of different alcoholic substrates show excellent results for compound **2** (Table 2), in accordance with the high accessibility of the framework to external molecules [26].

**Table 2:** Selective oxidation of several alcohols over μ-O-[FeTCPP]_2_·16DMF catalyst.

	Substrate	Oxidant	Product	TON ^a^	TOF (h^−1^) ^b^
1	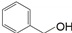	TBHP	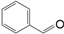	24	72
		PhI(OAc)_2_		23	50
2	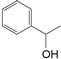	TBHP	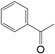	25	91
3	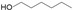	TBHP	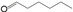	5	3
4	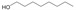	TBHP	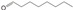	3	3

^a^ TON: Turnover number: mol subs.conv./mol cat; ^b^ TOF: mol subst.conv./mol cat. h).

**Figure 5:**
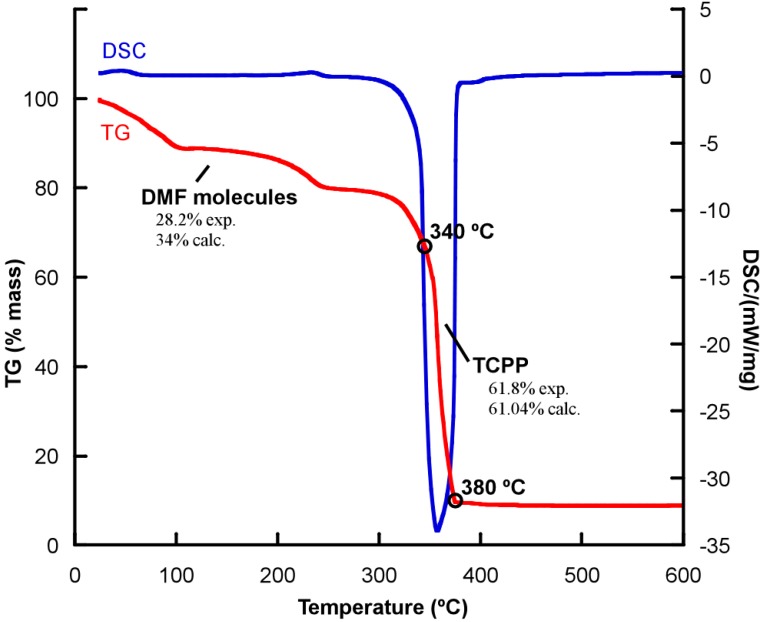
Thermogravimetric analysis for compound **1**.

The thermal behavior for compound **1** was studied by thermogravimetric analysis. The purity of the sample was also determined by powder X-ray diffraction (Figure S1). Compound **1** shows a three-stage mass loss from RT to 340 °C, assigned to the removal of the DMF molecules (28.2% weight loss) from the interporphyrinic cavities. Afterwards, a second mass loss occurs from 340 °C to 380 °C assigned to the porphyrinic units (61.8% weight loss) (Figure 5). The calcinations product has been identified using powder X-ray diffraction analysis, and consists of CuO (space group = *C*2/c, a = 4.685, b = 3.423, c = 5.132, β = 99.52) [29].

Thermal stability of compound **2** (Figure S2) has been reported elsewhere [26]. As for compound **1**, the H-bonded layers in **2** shows a high thermal stability, up to 360 °C.

#### IR Spectroscopy for Compounds **1** and **2**

**Figure 6:**
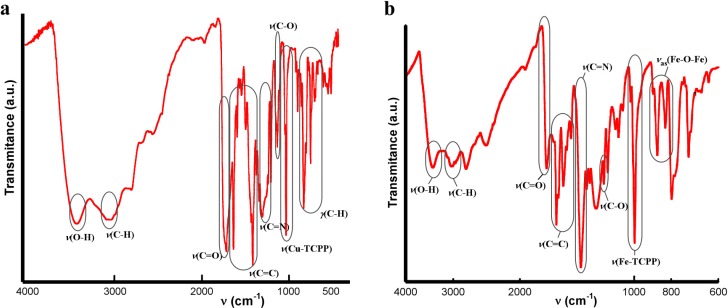
IR spectra for compounds (**a**) **1** and (**b**) **2**.

As shown in Figure 6, the observed IR absorptions for compounds **1** and **2** are the usual ones for TCPP-based porphyrin compounds. The bands around 3414 cm^−1^, 1705 cm^−1^ and 1320 cm^−1^ correspond to the peripheral carboxylic groups. The bands at 3030 cm^−1^, 1600–1450 cm^−1^ and 1380 cm^−1^ correspond to (C-H), (C=C) and (C-N) bonds respectively, and the metal-TCPP band appears at 1000 cm^−1^.

For compound **2**, the most significant IR spectral changes from the reactant [FeTCPP]Cl porphyrin to the final dimer account for the formation of the μ-oxo diiron(III) bond, with the appearance of two new strong absorptions at 870 and 827 cm^−1^. The antisymmetric stretching mode (*v*_3_), specifically the *v*_as_(Fe-O-Fe), of a linear or bent Fe-O-Fe system usually occurs in the range 900–800 cm^−1^ [30].

### 2.2. 1D Crystal Structures

With the purpose of obtaining monodimensional structures based on metalloporphyrin units, dipyridyl ligands were used as additional linkers. This way, compounds **3**, **4** and **5** were obtained using 4,4'-bipiridyne (bipy).

**Figure 7:**
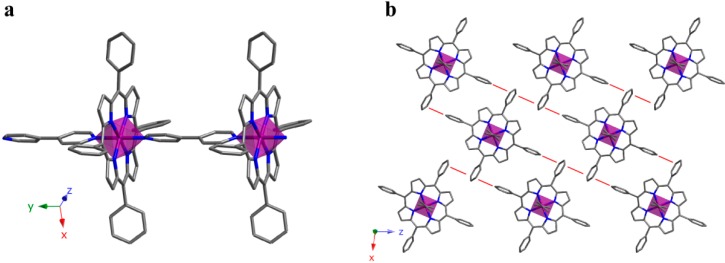
(**a**) 1D coordination polymers extending along the [010] direction and (**b**) view of a (101) plane for compound 3. (Fe: purple, C: grey, N: dark blue). π-π interactions are marked as red lines. H atoms have been omitted for clarity.

The crystal structure of compound **3** is based on FeTPP units, and consists of 1D coordination polymers extending along the [010] direction, where iron porphyrins are axially bonded to two bipy ligands (Figure 7a).

These coordination polymers crystallize as shown in Figure 7b, where the connections between chains take place through edge-to-face π stacking along the [10−1] direction (centroid-to-centroid distance of 3.662 Å, and angle of 83.94°). Additionally, there is a face-to-face π stacking along the [101] direction (centroid-to-centroid distance 5.067 Å and angle 0.02°). Therefore, the cohesion between 1D coordination polymers is based on a robust network of π bonds.

**Figure 8:**
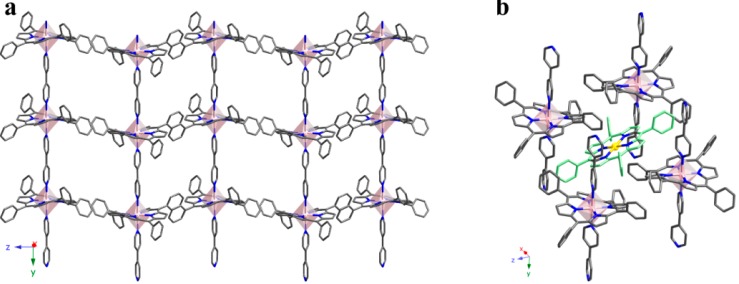
(**a**) View of the structure for compound **4** where the packing of the 1D polymers is observed; (**b**) Detail of the structure showing a single crystallization molecule of TPP connected to four 1D polymers through the π-bonding system. (Co(1): pink, Co(2): yellow, C: grey, N: dark blue and isolated porphyrin in green). H atoms have been omitted for clarity.

As compound **3**, compound **4** also consists of 1D polymers extending along the [010] direction. In this case, CoTPP units are axially bonded to two bipy ligands resulting in a porous coordination network (Figure 8a).

As shown in Figure 8b, in compound **4** isolated TPP units are located in the voids generated by the packing of these chains, due to an intricate system of π bonds. 78% of these isolated porphyrin units are metal-free, while the remaining 22% are metallated, in accordance with the chemical formula obtained by single crystal X-ray diffraction and elemental analysis: this is, [CoTPP(bipy)]·([CoTPP])_0.22_·(TPP)_0.78_. Therefore, most of the CoTPP units have lost the metal ion during the synthesis. Each isolated TPP unit is surrounded by four 1D polymers producing a dense network. As observed, there are multiple edge-to-face π-interactions stabilizing the crystal structure. These are robust interactions (distances from 2.45 Å to 2.97 Å, and angles from 73.96° to 89.16°), that are accompanied by weaker face-to-face ones (centroid-to-centroid distance 4.04 Å, and angle is 10.77°).

The bond distances and angles for the coordination spheres corresponding to both the Co^II^ ions in the chains and the Co^II^ ions in the crystallization complexes lie among typical values (Table S1). It must be pointed out that Co^II^ ions in the chains lie on a two-fold axis, and Co^II^ ions in the crystallization lie on an inversion center. As a result, both polyhedra are close to ideal.

**Figure 9:**
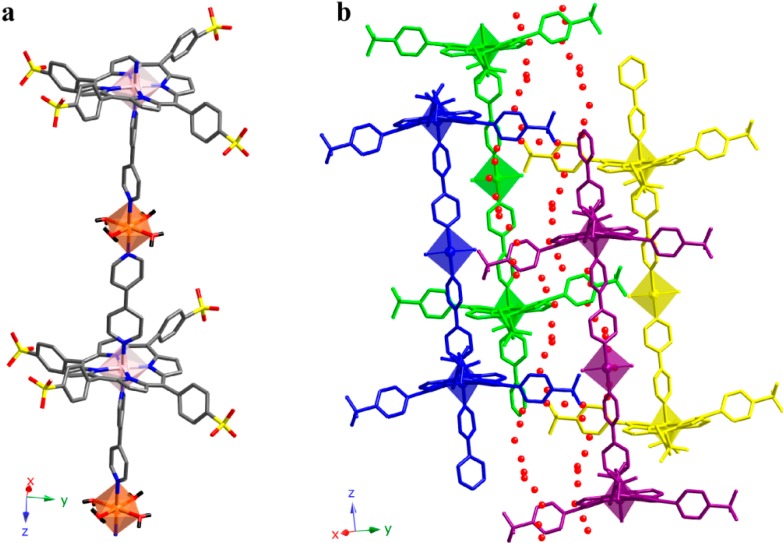
(**a**) Detail of the structure showing the extension of the 1D polymers and (**b**) relative position of the chains for compound **5**. Crystallization molecules of water are shown in red. (Co(1) (TPPS): pink, Co(2): orange, C: grey, N: dark blue, O: red, S: yellow). H atoms have been omitted for clarity.

Compound **5** exhibits some similarities with compounds **3** and **4**. In fact, compound **5** also consists of 1D polymers where CoTPPS units are axially bonded to bipy ligands. However, the extension of the 1D polymers for compound **5** consists of the link between alternating metal centers along the [001] direction. These links take place through the bipy ligands according to the bipy-CoTPPS-bipy-Co(H_2_O)_4_-fashion (Figure 9a).

Compound **5** exhibits a robust system of hydrogen bonds that reinforce the stability of the framework (Table S2). This way, each chain is surrounded by another four, and multiple hydrogen bonds are formed between the coordination molecules of water and the terminal SO_3_ groups of the TPPS molecules.

As observed in Figure 9b, the relative position of the chains gives rise to cavities where crystallization molecules of water are located. These molecules form a disordered chain along the [001] direction, reinforcing the robustness of the hydrogen bond system.

The analysis of monodimensional compounds show that the interactions between the chains make the difference. Using TPP molecules, with a benzene ring in the *meso* positions, we observed that the interaction is given by weak π type bonds, allowing the introduction of TPP isolated molecules between chains (compound **4**). However, by introducing SO_3_ groups in the *meso* position of the TPPS porphyrin, as occurs in compound **5**, the resulting H-bond system is stronger. 

Thermal stability for compounds **3**, **4** and **5** has been reported elsewhere [24,25]. As shown in Figures S3–S5 the compounds **3**, **4** and **5** were thermally stable up to 290 °C, 230 °C and 370 °C, respectively, confirming the robustness of the H-bond system observed for compound **5**.

#### IR Spectroscopy for Compounds **3**, **4** and **5**

**Figure 10:**
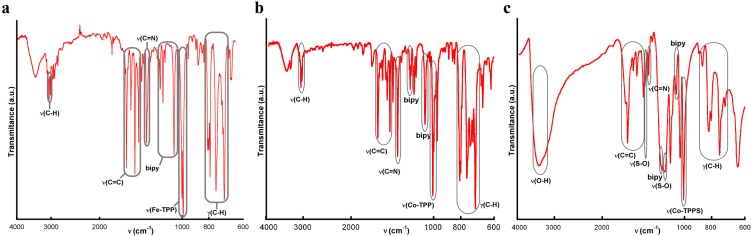
IR spectra for compounds (**a**) **3**; (**b**) **4** and (**c**) **5**.

As shown in Figure 10, the IR spectra for compounds **3**, **4** and **5** were quite similar. The absorptions observed between 3052 and 2964 cm^−1^ are assigned to the C(sp^2^)-H vibrations. The bands at 1600–1440 cm^−1^ and 1350 cm^−1^ were assigned to the C=C stretching mode and C-N stretching vibration, respectively. The vibration modes of the bipyridine molecule appear at 1210 and 1070 cm^−1^ and the metal-porphyrin vibration at 1000 cm^−1^. Finally, the bands in the range of 860–700 cm^−1^ were assigned to the H out of plane bending of the C-H bond. Additionally, compound **5** exhibit some characteristic bands at 3397 cm^−1^ and 1174 cm^−1^ corresponding to the water molecules and the SO_3_ groups, respectively.

### 2.3. 2D Crystal Structure

Increasing another step in dimensionality we have obtained a 2D SCF. The crystal structure of compound **6** is constructed with FeTCPP complexes containing carboxylic groups on the peripheral position of the porphyrin. The iron atom is octahedrally coordinated to four N atoms on the porphyrin equatorial plane while the axial positions are occupied by two O atoms that belong to the carboxylate groups of adjacent porphyrin units. Table S3 summarizes the most significant bond parameters for compounds **6**. As observed, the values lie among the typical ones for octahedral iron(III) ions.

**Figure 11:**
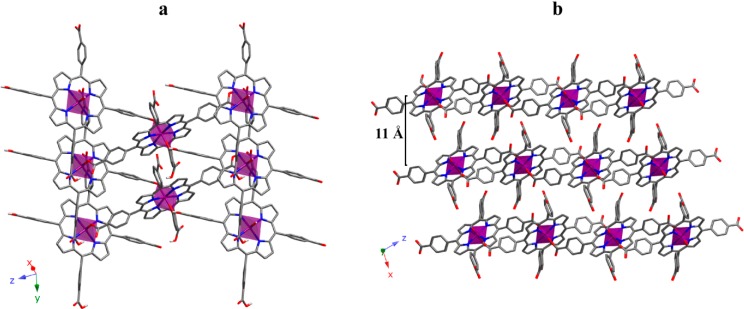
(**a**) View of a 2D layer and (**b**) projection of the 3D supramolecular array for compound **6**. (Fe: purple, C: grey, N: dark blue, O: red). H atoms have been omitted for clarity.

Taking the structural fragment of a porphyrin unit as reference, the polymer extends through the formation of four new bonds with adjacent porphyrin groups. Thus, the reference porphyrin uses its own O atoms to connect with another two porphyrin units. This is, each unit is connected to four equivalent units, and the result is a 2D array (Figure 11a). This planar array is based on the fact that octahedral spheres are rotated forming planes that are perpendicular to the [10–1] direction, the extension of the structure taking place through coordination bonds. Additionally, there are several hydrogen bonds (Table S4) between atoms on the same plane, which reinforce the robustness of the 2D moiety.

It is worth mentioning that this 2D framework is chiral. Undoubtedly, the presence of 2D chiral layer is a remarkable fact, since this property is not usual for structures based on porphyrin blocks exhibiting *D*_4h_ molecular symmetry [31]. Obviously, the fact that the structure extends through both axial sites and two of the equatorial ones is responsible for the later.

As shown in Figure 11b, packing on the planes along the [10–1] direction gives rise to the 3D framework. These planes are chemically connected through strong hydrogen bonds between carboxylate groups. As a consequence of this packing fashion, the 3D framework keeps the chirality observed for the 2D array.

Crystal structure refinement for compound **6** shows a Flack parameter value of 0.454(4), indicating a partial mixture of each enantiomers. Additionally, the diffraction data (intensity statistics and systematic absences), as well as the phase determination and structure solution processes, were consistent only with the non-centrosymmetric space group *P*2_1_, with two screw-related porphyrin molecules per unit-cell. The resulting structural model reveals a high degree of pseudosymmetry, resembling the centrosymmetric space group *P*2_1_/*c*. Additional attempts to solve and refine this structure in *P*2_1_/*c* have failed.

According to the obtained 2D compound and the examples found in literature [32], the way in which the metalloporphyrinic units are connected is crucial for the growth of the crystal structures. While in compound **6** the connection takes place between the *meso* and axial positions of the metalloporphyrin resulting in compact layers, when the metalloporphyrins are only connected by the *meso* positions, the frameworks are more accessible to external molecules.

Table 3 shows a matrix showing the combinations of metal ions and porphiryns used in this work. Additionally, Table 3 also summarizes the number of structures found on the Cambridge Structural Database (CSD v5.33) [33] for each combination. As observed, Fe-based compounds are the most investigated. However, no Fe-TPPS compounds have been reported so far. On the other hand, Cu-based compounds are still scarce, and as for iron, no Cu-TPPS compounds have been reported. In fact, TPPS porphiryn has produced complexes just with cobalt (compound **5** being one of the two existing ones).

**Table 3:** Reported crystal structures with TPP, TCPP and TPPS porphyrins with Fe, Co and Cu in the Cambridge Structural Database (CSD).

	TPP	TCPP	TPPS
Fe	compound 3 (+273)	compounds 2 and 6 (+12)	no compounds
Co	compound 4 (+79)	(+7)	compound 5 (+1)
Cu	(+12)	compound 1 (+9)	no compounds

#### IR Spectroscopy for Compound **6**

**Figure 12:**
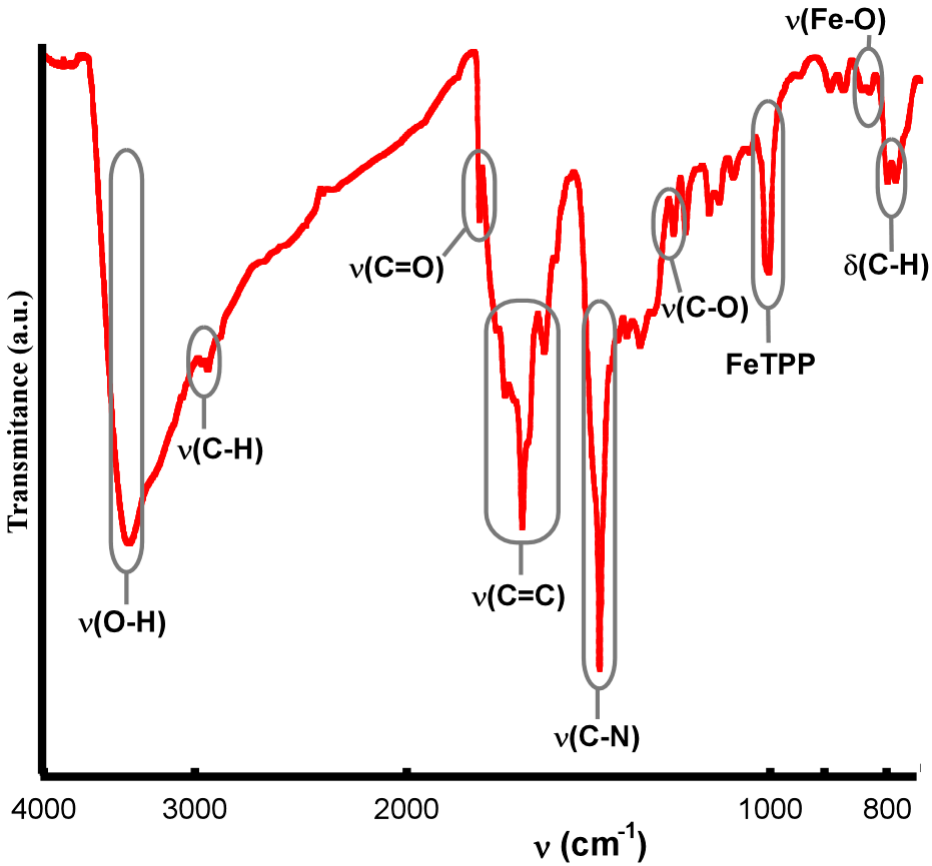
IR spectrum for compound **6**.

IR data for compound **6** show significant differences between the free ligand TCPP and the metallated [FeTCPP] compound. The N-H bond stretching and bending frequencies of TCPP located at 3200 cm^−1^ and 970 cm^−1^ disappeared when iron ion was inserted into the porphyrin ring, and a characteristic Fe-TCPP band appears at 1000 cm^−1^, which indicates the formation of an iron porphyrin compound [34]. The bands at about 3415 cm^−1^, 1740 cm^−1^ and 1200 cm^−1^ were assigned to the O-H, C=O and C-O bonds of the carboxylic groups, respectively. The band at 2940 cm^−1^ was assigned to the C-H bond of the benzene and pyrrole rings. The bands at 1690–1540 cm^−1^ and 1380 cm^−1^ were assigned to C=C stretching mode and the C-N stretching vibration, respectively. Finally, the band at 790 cm^−1^ was assigned to the H out of plane bending of the C-H bond (Figure 12).

## 3. Experimental Section

### 3.1. Materials

All solvents and chemicals were used as received from reliable commercial sources. The *meso*-tetra(4-carboxyphenyl)porphyrin (TCPP), *meso*-tetraphenyl porphine-4,4',4'',4'''-tetrasulfonic acid tetrasodium salt (TPPS), *meso*-tetraphenyl porphine cobalt(II) (CoTPP) and *meso*-tetraphenyl-porphine iron(III) chloride ([FeTPP]Cl) porphyrins were purchased from Sigma-Aldrich Co. (St. Louis, MO, USA) The cooper(II) *meso*-tetra(4-carboxyphenyl)porphine (CuTCPP) was purchased from Frontier Scientific. (Logan, UT, USA) Cobalt(II) nitrate hexahydrate 99%, iron(II) chloride 98%, 4,4'-bipyridine 98% (bipy), and the solvents *N*,*N*-dimethylformamide 99.8% (DMF) and ethanol 96% were purchased from Sigma-Aldrich Co.

### 3.2. Synthesis

Synthesis of compounds **2** [26], **3** [25], **4**, **5** [24] and **6** [23] were previously reported.

#### Synthesis of [CuTCPP]·6DMF (**1**)

Copper(II) *meso*-tetra(4-carboxyphenyl)porphine (8.8 mg, 0.01 mmol) and fumaric acid (9.9 mg, 0.06 mmol) were dissolved in DMF (5 mL) in a small capped vial, sonicated to ensure homogeneity and heated to 80 °C for 72 h, followed by 72 h of evaporation in a crystallizing dish, yielding diffraction quality fibrous red crystals. ν_max_/cm^−1^: 3403 (C(sp2)H), 2770 (OH), 1390–1280 (C=O). 1600−1450 (CC), 1320 (CO), 1380 (CN), 1006 (CuTCPP), 790–600 (CH). Found: C, 59.91; H, 5.17; N, 9.54; O, 18.40. Calc. for C_66_H_68_CuN_10_O_14_: C, 61.50; H, 5.32; N, 10.87; O, 17.38.

### 3.3. X-ray Structure Determination

X-ray structure determinations for compounds **2** [26], **3** [25], **4**, **5** [24] and **6** [23] were previously reported. For compound **1** single-crystals with dimensions given in Table 1 were selected under polarizing microscope and mounted on MicroMounts™. Single-crystal X-ray diffraction data were collected at 100 K on an Agilent Technologies SuperNova single source diffractometer with Cu-Kα radiation (λ = 1.54184 Å). Data frames were processed (unit cell determination, intensity data integration, correction for Lorentz and polarization effects [35], and analytical absorption correction) using the CrysAlisPro software package [36]. The structure of compounds **1** was solved in the monoclinic *C*2/c space group with Superflip program [37], which allowed us to obtain the position of Cu atoms, as well as nitrogen, oxygen and some of the carbon atoms of the porphyrin. The refinement of the crystal structure was performed by full matrix least-squares based on *F^2^*, using the SHELXL-97 program [38] obtaining the remaining carbon atoms. Anisotropic thermal parameters were used for all non-hydrogen atoms (Figure S6). All the hydrogen atoms connected to the aromatic rings (C-H 0.93 Ǻ) and to the DMF molecules (C-H 0.96 Ǻ) were fixed geometrically, and were refined using a riding model with common isotropic displacements. Bond distances and angles, atomic coordinates, anisotropic thermal parameters and hydrogen atom coordinates are given in Tables S5–S8.

### 3.4. Physicochemical Characterization Techniques

The IR spectra were collected on a JASCO FT/IR-6100 spectrometer at room temperature in the range of 4000–600 cm^−1^, in KBr pellets (1% of the sample). C, H, N, S and O elemental analyses were measured using a Euro EA 3000 elemental analyzer. The thermal analyses were carried out in air atmosphere using a NETZSCH STA 449F3 instrument or a SDT 2960 Simultaneous DSC-TGA TA Instruments. A crucible containing approximately 10 mg of sample was heated at 5 °C·min^−^^1^ in the temperature range 30–600 °C.

## 4. Conclusions

This work provides some examples confirming that the chemical features of the porphyrins and the nature of the central metals are of great importance on the extension of the 2D metalloporphyrin based Solid Coordination Frameworks (SCFs). While the structures extended just through *meso* positions are accessible to external molecules, when axial positions are involved the as-formed layers are much more compact. The *meso* substituents on porphyrins of 1D compounds are responsible on the framework robustness, as well as for 0D compounds where the interporphyrin contacts and solvent molecules interactions stabilize the framework. Additionally, coordination and crystallization molecules provide the crystal structures with cohesion forces (H bonds and π interactions) that reinforce the robustness of the arrays. On the other hand, even if, generally speaking, high dimensional arrays are intended, we have observed excellent catalytic activity in a 0D SCF, as a result of the high accessibility to external molecules. This confirms that bioinspired metalloporphyrins-based systems can be applied for application mimicking their natural functions.

## Supplementary Materials

Supplementary materials can be accessed at: http://www.mdpi.com/1420-3049/20/04/6683/s1.
