# Supplementary material for: Coordination and Crystallization Molecules: Their Interactions Affecting the Dimensionality of Metalloporphyrinic SCFs
Source: Molecules. 2015 Apr 15;20(4):6683–99. doi: 10.3390/molecules20046683 (PMC6272730; doi:10.3390/molecules20046683)
Supplement: Supplementary file 1 [file molecules-20-06683-s001.pdf]

Supplementary Materials

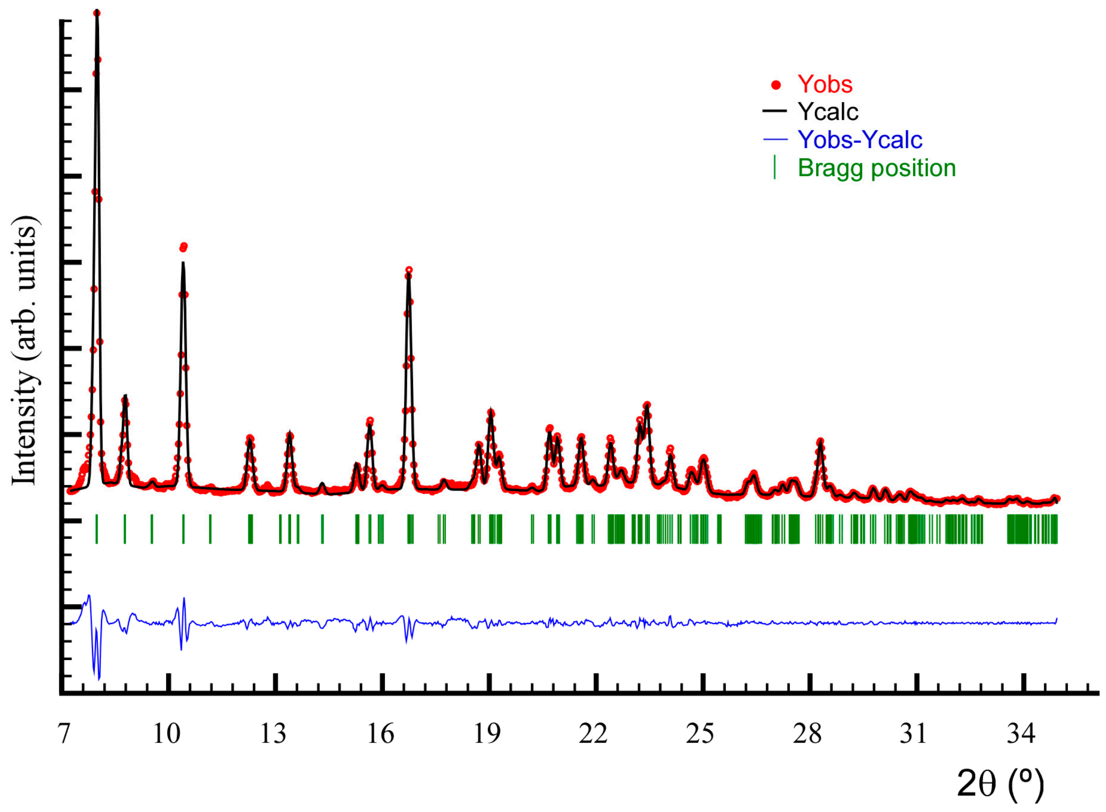

Figure S1. X-ray full profile analysis for compound **1**.

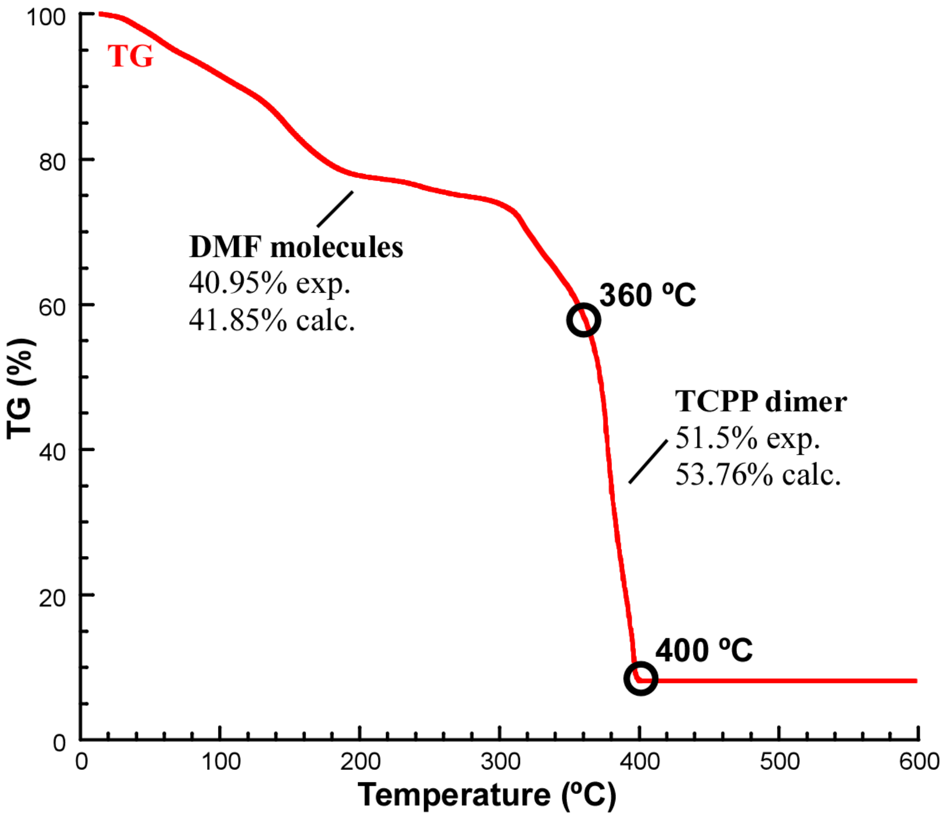

Figure S2. Thermogravimetric analysis for compound **2**.

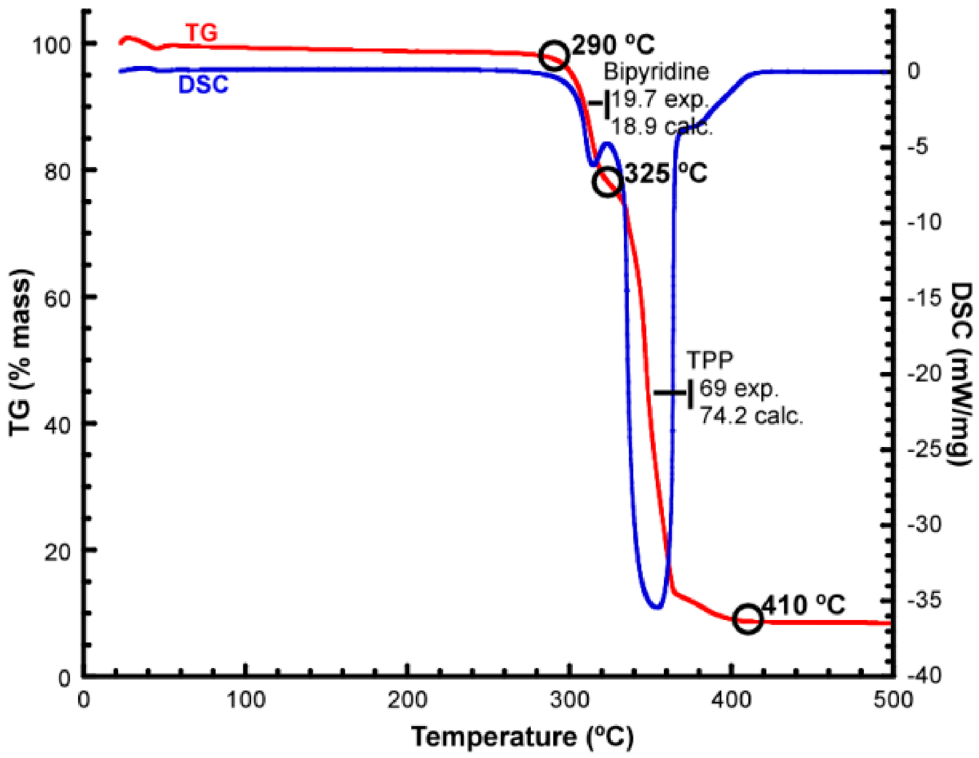

Figure S3. Thermogravimetric analysis for compound **3**.

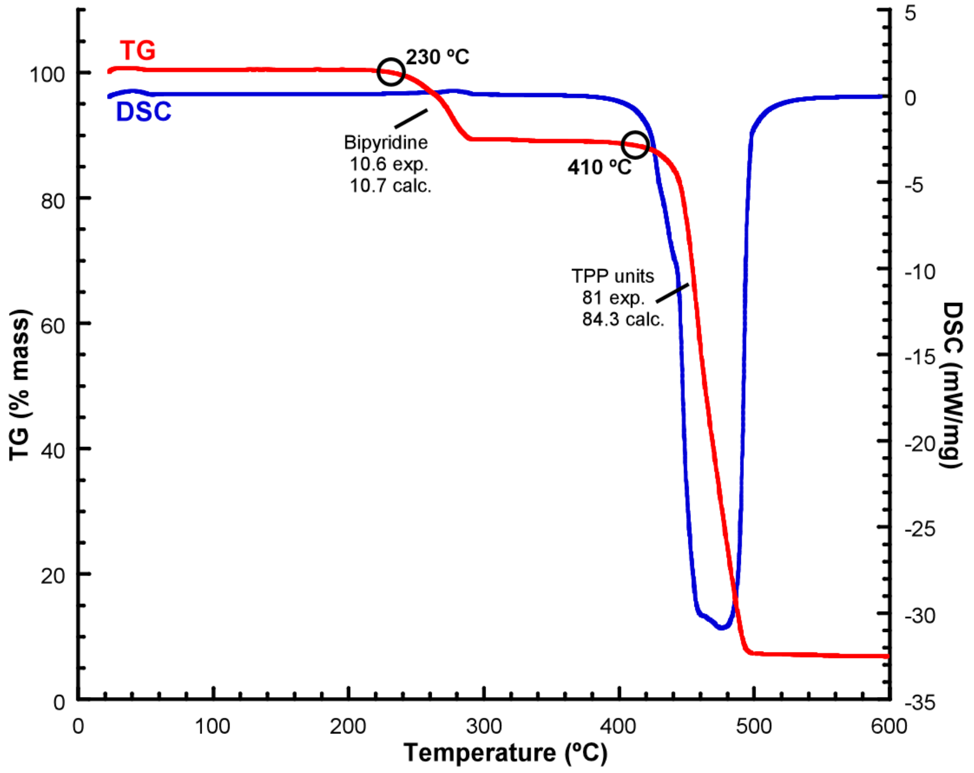

Figure S4. Thermogravimetric analysis for compound **4**.

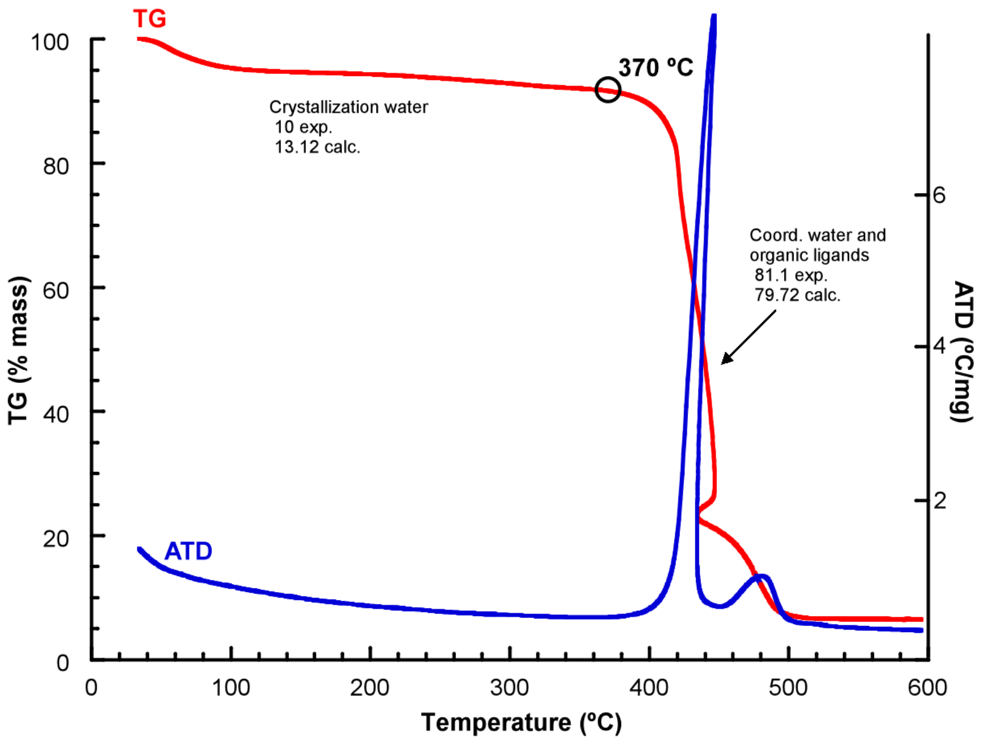

Figure S5. Thermogravimetric analysis for compound **5**.

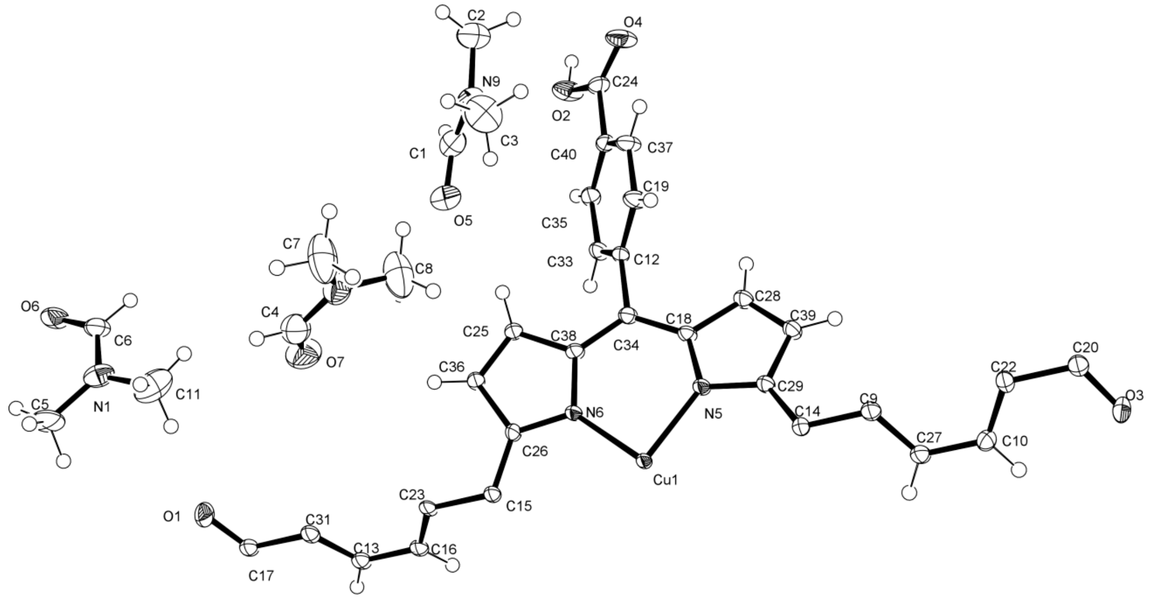

Figure S6. ORTEP detail for compound **1**. The thermal ellipsoids correspond to 50% probability.

Table S1. Most significant angles (°) and distances (Å) for compounds **4** and **5** (distances in bold).

		Co(1). CoN_6 Octahedra				Co(2). CoN_4 Square Planar		
	**Co1**	**N1**	**N2**	**N3**	**N4**	**Co2**	**N5**	**N6**
Compound **4**	**N4**	89.84(4)	89.89(4)	180	**2.296(2)**	**N6**	90.38(5)	**2.055(1)**
	**N3**	90.16(4)	90.11(4)	**2.357(2)**		**N5**	**2.032(1)**	
	**N2**	90.21(6)	**1.962(1)**					
	**N1**	**1.966(1)**						
		Co(1). CoN_6 Octahedra				Co(2). $CoN_2(H_2O)_4$ Octahedra		
	**Co1**	**N1**	**N2**			**Co2**	**O4**	**N3**
Compound **5**	**N2**	88.20(7)	**1.976(4)**			**N3**	90.76(6)	**2.137(4)**
	**N1**	**1.963(3)**				**O4**	**2.092(3)**	

Table S2. Hydrogen bond parameters for compound **5**.

D-H	A	D-H (Å)	H···A (Å)	O-H···A (°)
O(4)-H(20)	$O(3)^{i}$	0.81(3)	1.93(3)	173(4)
O(4)-H(21)	$O(1)^{ii}$	0.82(4)	1.89(4)	178(4)

Symmetry codes (i) −1/2 + x, 1/2 + y, 1/2 + z; (ii) −1/4 − y, −1/4 + x, 3/4 − z

Table S3. Most significant angles (°) and distances (Å) for compounds **6** (distances in bold).

		Fe(1). FeN_4O_2 Octahedra					
	**Fe**	**N1**	**N2**	**N3**	**N4**	**O1**	**O6**
Compound **6**	**O6**	85.44(10)	89.98(10)	86.91(11)	84.09(10)	178.34(11)	**2.220(3)**
	**O1**	95.62(11)	88.74(11)	92.03(11)	97.18(11)	**1.988(2)**	
	**N4**	90.15(11)	174.07(13)	89.35(11)	**2.060(3)**		
	**N3**	172.34(13)	90.17(11)	**2.039(3)**			
	**N2**	89.54(11)	**2.067(3)**				
	**N1**	**2.030(3)**					

Table S4. Intra- and interlayer hydrogen bond parameters for compound **6**.

D-H	A	D-H (Å)	H···A (Å)	O-H···A (°)
O(4)-H(40)	$O(2)^{i}$	0.84	1.78(4)	171(4)
O(5)-H(50)	$O(3)^{ii}$	0.84	2.00(4)	144(2)
O(8)-H(80)	$O(2)^{iii}$	0.84	1.91(5)	169(3)

Symmetry codes (i) 2 − x,−3/2 + y, −z; (ii) 2 − x, 1/2 + y,1 – z; (iii) 1 − x, 1/2 + y, −z

Table S5. Bond distances (Å) and angles (°) for compound **1**.

Distances			
Cu(1)-N(6)	1.998(1)	O(6)-C(6)	1.235(4)
Cu(1)-N(6)[i]	1.998(1)	N(1)-C(11)	1.450(4)
Cu(1)-N(5)[i]	2.003(1)	C(5)-H(5A)	0.9600
Cu(1)-N(5)	2.003(1)	C(5)-H(5C)	0.9600
O(1)-C(17)	1.268(2)	C(11)-H(11A)	0.9600
O(2)-C(24)	1.317(3)	C(11)-H(11C)	0.9600
O(2)-H(2)	0.8199	N(7)-C(4)	1.337(4)
O(3)-C(20)	1.272(2)	N(7)-C(7)	1.460(5)
O(4)-C(24)	1.207(3)	C(7)-H(7A)	0.9600
N(5)-C(29)	1.369(3)	C(7)-H(7C)	0.9600
N(5)-C(18)	1.371(3)	C(8)-H(8B)	0.9600
N(6)-C(26)	1.372(3)	C(13)-C(16)	1.382(3)
N(6)-C(38)	1.380(3)	C(13)-H(13)	0.9300
C(9)-C(27)[i]	1.400(3)	C(14)-C(29)[i]	1.404(3)
C(9)-C(27)	1.400(3)	C(15)-C(26)	1.400(3)
C(9)-C(14)	1.491(4)	C(16)-C(23)	1.403(3)
C(10)-C(27)	1.378(3)	C(17)-O(1)[i]	1.268(2)
C(10)-C(22)	1.392(3)	C(18)-C(28)	1.447(3)
C(10)-H(10)	0.9300	C(19)-H(19)	0.9300
C(12)-C(19)	1.397(3)	C(20)-C(22)	1.484(4)
C(12)-C(33)	1.398(3)	C(23)-C(16)[i]	1.403(3)
C(12)-C(34)	1.487(3)	C(25)-C(36)	1.354(3)
C(12)-C(34)	1.487(3)	C(25)-H(25)	0.9300
C(13)-C(31)	1.396(3)	C(27)-H(27)	0.9300
C(14)-C(29)	1.404(3)	C(28)-H(28)	0.9300
C(15)-C(26)[i]	1.400(3)	C(31)-C(13)[i]	1.396(3)
C(15)-C(23)	1.488(4)	C(33)-H(33)	0.9300
C(16)-H(16)	0.9300	C(35)-C(40)	1.390(3)
C(17)-C(31)	1.488(4)	C(36)-H(36)	0.9300
C(19)-C(37)	1.388(3)	C(37)-H(37)	0.9300
C(20)-O(3)[i]	1.272(2)	O(5)-C(1)	1.230(4)
C(22)-C(10)[i]	1.392(3)	N(9)-C(3)	1.439(5)
C(24)-C(40)	1.497(3)	C(1)-H(1)	0.9300
C(25)-C(38)	1.438(3)	C(2)-H(2B)	0.9600
C(26)-C(36)	1.444(3)	C(3)-H(3A)	0.9600
C(28)-C(39)	1.358(3)	C(3)-H(3C)	0.9600
C(29)-C(39)	1.440(3)	N(1)-C(6)	1.320(4)
C(33)-C(35)	1.385(3)	N(1)-C(5)	1.452(4)
C(34)-C(38)	1.395(3)	C(5)-H(5B)	0.9600
C(35)-H(35)	0.9300	C(6)-H(6)	0.9300
C(37)-C(40)	1.394(3)	C(11)-H(11B)	0.9600
C(39)-H(39)	0.9300	O(7)-C(4)	1.204(4)
N(9)-C(1)	1.329(4)	N(7)-C(8)	1.435(5)
N(9)-C(2)	1.451(4)	C(4)-H(4)	0.9300
C(2)-H(2A)	0.9600	C(7)-H(7B)	0.9600
C(2)-H(2C)	0.9600	C(8)-H(8A)	0.9600
C(3)-H(3B)	0.9600	C(8)-H(8C)	0.9600

Table S5. *Cont.*

Angles			
N(6)-Cu(1)-N(6)^{i}	89.69(1)	C(10)^{i}-C(22)-C(20)	120.51(1)
N(6)-Cu(1)-N(5)^{i}	175.59(7)	C(10)-C(22)-C(20)	120.51(1)
N(6)^{i}-Cu(1)-N(5)^{i}	90.44(8)	C(16)-C(23)-C(16)^{i}	118.5(3)
N(6)-Cu(1)-N(5)	90.44(8)	C(16)-C(23)-C(15)	120.86(1)
N(6)^{i}-Cu(1)-N(5)	175.59(7)	C(16)^{i}-C(23)-C(15)	120.86(1)
N(5)^{i}-Cu(1)-N(5)	89.96(1)	O(4)-C(24)-O(2)	124.0(2)
C(24)-O(2)-H(2)	109.4	O(4)-C(24)-C(40)	123.3(2)
C(29)-N(5)-C(18)	106.2(1)	O(2)-C(24)-C(40)	112.6(2)
C(29)-N(5)-Cu(1)	126.8(1)	C(36)-C(25)-C(38)	107.0(2)
C(18)-N(5)-Cu(1)	126.3(1)	C(36)-C(25)-H(25)	126.5
C(26)-N(6)-C(38)	106.1(1)	N(6)-C(26)-C(15)	125.2(2)
C(26)-N(6)-Cu(1)	127.1(1)	N(6)-C(26)-C(36)	109.9(1)
C(38)-N(6)-Cu(1)	126.3(1)	C(15)-C(26)-C(36)	124.7(2)
C(27)^{i}-C(9)-C(27)	118.5(3)	C(10)-C(27)-C(9)	120.6(2)
C(27)^{i}-C(9)-C(14)	120.8(1)	C(10)-C(27)-H(27)	119.7
C(27)-C(9)-C(14)	120.8(1)	C(9)-C(27)-H(27)	119.7
C(27)-C(10)-C(22)	120.5(2)	C(39)-C(28)-C(18)	106.9(1)
C(27)-C(10)-H(10)	119.7	C(39)-C(28)-H(28)	126.6
C(22)-C(10)-H(10)	119.7	C(18)-C(28)-H(28)	126.6
C(19)-C(12)-C(33)	118.5(2)	N(5)-C(29)-C(14)	125.2(2)
C(19)-C(12)-C(34)	120.9(2)	N(5)-C(29)-C(39)	110.4(1)
C(33)-C(12)-C(34)	120.7(2)	C(14)-C(29)-C(39)	124.3(2)
C(16)-C(13)-C(31)	120.0(2)	C(13)-C(31)-C(13)^{i}	119.9(3)
C(16)-C(13)-H(13)	120.0	C(13)-C(31)-C(17)	120.1(1)
C(31)-C(13)-H(13)	120.0	C(13)^{i}-C(31)-C(17)	120.1(1)
C(29)-C(14)-C(29)^{i}	123.1(3)	C(35)-C(33)-C(12)	120.7(2)
C(29)-C(14)-C(9)	118.5(1)	C(35)-C(33)-H(33)	119.6
C(29)^{i}-C(14)-C(9)	118.5(1)	C(12)-C(33)-H(33)	119.6
C(26)^{i}-C(15)-C(26)	122.9(3)	C(38)-C(34)-C(18)	123.7(2)
C(26)^{i}-C(15)-C(23)	118.6(1)	C(38)-C(34)-C(12)	117.6(1)
C(26)-C(15)-C(23)	118.6(1)	C(18)-C(34)-C(12)	118.8(1)
C(13)-C(16)-C(23)	120.8(2)	C(33)-C(35)-C(40)	120.4(2)
C(13)-C(16)-H(16)	119.6	C(33)-C(35)-H(35)	119.8
C(23)-C(16)-H(16)	119.6	C(40)-C(35)-H(35)	119.8
O(1)^{i}-C(17)-O(1)	123.0(3)	C(25)-C(36)-C(26)	107.1(1)
O(1)^{i}-C(17)-C(31)	118.6(1)	C(25)-C(36)-H(36)	126.5
O(1)-C(17)-C(31)	118.6(1)	C(26)-C(36)-H(36)	126.5
N(5)-C(18)-C(34)	125.6(2)	C(19)-C(37)-C(40)	120.0(2)
N(5)-C(18)-C(28)	109.8(1)	C(19)-C(37)-H(37)	120.0
C(34)-C(18)-C(28)	124.6(2)	C(40)-C(37)-H(37)	120.0
C(37)-C(19)-C(12)	120.9(2)	N(6)-C(38)-C(34)	125.4(2)
C(37)-C(19)-H(19)	119.5	N(6)-C(38)-C(25)	109.7(1)
C(12)-C(19)-H(19)	119.5	C(34)-C(38)-C(25)	124.6(2)
O(3)^{i}-C(20)-O(3)	123.4(3)	C(28)-C(39)-C(29)	106.7(1)
O(3)^{i}-C(20)-C(22)	118.3(1)	C(28)-C(39)-H(39)	126.7
O(3)-C(20)-C(22)	118.3(1)	C(29)-C(39)-H(39)	126.7
C(10)^{i}-C(22)-C(10)	119.2(3)		

Symmetry code: (i) −x, y, −z − 3/2

Table S6. Fractional atomic coordinates (×10[4]) and equivalent thermal factors (×10[3]) for compound **1**.

Atoms	X	Y	Z	U_{eq}, Å^2
Cu(1)	0	−625(1)	−7500	13(1)
O(1)	−273(1)	−5042(1)	−7001(2)	29(1)
O(2)	−2598(1)	−1086(1)	−15,336(2)	36(1)
O(3)	314(1)	3789(1)	−7663(2)	26(1)
O(4)	−2792(1)	−290(1)	−14,104(3)	36(1)
N(5)	−380(1)	13(1)	−8614(2)	15(1)
N(6)	−396(1)	−1263(1)	−8457(2)	15(1)
C(9)	0	1591(1)	−7500	18(1)
C(10)	324(1)	2533(1)	−7804(3)	19(1)
C(12)	−1351(1)	−630(1)	−11,114(3)	16(1)
C(13)	242(1)	3786(1)	−8333(3)	19(1)
C(14)	0	920(1)	−7500	16(1)
C(15)	0	−2171(1)	−7500	15(1)
C(16)	238(1)	−3164(1)	−8347(3)	18(1)
C(17)	0	−4770(1)	−7500	20(1)
C(18)	−736(1)	−79(1)	−9737(3)	16(1)
C(19)	−1660(1)	−301(1)	−10,708(3)	23(1)
C(20)	0	3518(1)	−7500	19(1)
C(22)	0	2850(1)	−7500	17(1)
C(23)	0	−2841(1)	−7500	16(1)
C(24)	−2542(1)	−656(1)	−14,223(3)	22(1)
C(25)	−1002(1)	−1729(1)	−9510(3)	17(1)
C(26)	−359(1)	−1870(1)	−8175(3)	15(1)
C(27)	324(1)	1913(1)	−7810(3)	18(1)
C(28)	−878(1)	482(1)	−10,544(3)	18(1)
C(29)	−309(1)	619(1)	−8613(3)	16(1)
C(31)	0	−4100(1)	−7500	19(1)
C(33)	1438(1)	−971(1)	−12,559(3)	18(1)
C(34)	−941(1)	−625(1)	−10,025(3)	16(1)
C(35)	−1822(1)	−990(1)	−13,550(3)	19(1)
C(36)	−738(1)	−2163(1)	−8824(3)	18(1)
C(37)	−2043(1)	−306(1)	−11,723(3)	24(1)
C(38)	−785(1)	−1170(1)	−9333(3)	16(1)
C(39)	613(1)	914(1)	−9845(3)	18(1)
C(40)	−2127(1)	−658(1)	−13,142(3)	19(1)
O(5)	−1963(1)	−2137(1)	−10,576(3)	46(1)
N(9)	−2521(1)	−1600(1)	−10,505(3)	40(1)
C(1)	−2309(1)	−1977(1)	−11,191(4)	37(1)
C(2)	−2937(1)	−1473(2)	−11,270(6)	62(1)
C(3)	2357(1)	−1346(2)	−8906(5)	59(1)
O(6)	−1743(1)	−6027(1)	−7477(2)	35(1)
N(1)	−1277(1)	−5462(1)	−5775(3)	33(1)
C(5)	−1059(1)	−5976(2)	−4941(4)	44(1)
C(6)	−1602(1)	−5533(1)	−6969(3)	30(1)
C(11)	−1124(1)	−4865(2)	−5287(5)	54(1)
O(7)	−1119(1)	−3430(1)	−6215(3)	54(1)
N(7)	−1424(1)	−2742(1)	−4923(3)	40(1)
C(4)	−1253(1)	−3272(2)	−5076(4)	42(1)
C(7)	−1583(2)	−2596(2)	−3500(5)	65(1)
C(8)	−1448(2)	−2281(2)	−6142(5)	66(1)

$U_{eq} = 1/3[U_{11}(aa^*)^2 + U_{22}(bb^*)^2 + U_{33}(cc^*)^2 + 2U_{13}aca^*c^* \cos\beta]$.

Table S7. Anisotropic displacement parameters ($A^2 \times 10^3$) for compound **1**.

Atoms	U11	U22	U33	U23	U13	U12
Cu(1)	11(1)	11(1)	16(1)	0	−2(1)	0
O(1)	29(1)	15(1)	46(1)	−1(1)	12(1)	−2(1)
O(2)	20(1)	45(1)	34(1)	−12(1)	−13(1)	7(1)
O(3)	26(1)	15(1)	35(1)	2(1)	2(1)	−5(1)
O(4)	17(1)	44(1)	41(1)	−8(1)	−5(1)	9(1)
N(5)	13(1)	15(1)	16(1)	−1(1)	−1(1)	−1(1)
N(6)	12(1)	15(1)	16(1)	1(1)	−2(1)	1(1)
C(9)	13(2)	20(2)	16(1)	0	−2(1)	0
C(10)	17(1)	18(1)	20(1)	1(1)	1(1)	−3(1)
C(12)	13(1)	15(1)	17(1)	4(1)	−3(1)	1(1)
C(13)	15(1)	17(1)	20(1)	−1(1)	−4(1)	1(1)
C(14)	15(1)	15(1)	16(1)	0	2(1)	0
C(15)	14(1)	16(1)	13(1)	0	−2(1)	0
C(16)	14(1)	18(1)	17(1)	1(1)	−3(1)	0(1)
C(17)	15(2)	16(2)	24(2)	0	−3(1)	0
C(18)	14(1)	16(1)	16(1)	−1(1)	0(1)	4(1)
C(19)	17(1)	24(1)	25(1)	−5(1)	−3(1)	3(1)
C(20)	20(2)	18(2)	16(1)	0	1(1)	0
C(22)	21(2)	15(1)	13(1)	0	0(1)	0
C(23)	10(1)	15(1)	18(1)	0	−7(1)	0
C(24)	15(1)	27(1)	23(1)	2(1)	−1(1)	−2(1)
C(25)	11(1)	17(1)	19(1)	1(1)	−3(1)	−2(1)
C(26)	15(1)	12(1)	17(1)	−1(1)	0(1)	−1(1)
C(27)	16(1)	18(1)	19(1)	−2(1)	1(1)	1(1)
C(28)	17(1)	19(1)	17(1)	2(1)	−2(1)	2(1)
C(29)	13(1)	15(1)	17(1)	1(1)	−1(1)	2(1)
C(31)	15(2)	16(2)	19(2)	0	−7(1)	0
C(33)	17(1)	18(1)	18(1)	2(1)	0(1)	2(1)
C(34)	14(1)	16(1)	17(1)	−1(1)	2(1)	1(1)
C(35)	19(1)	19(1)	17(1)	0(1)	−2(1)	0(1)
C(36)	16(1)	15(1)	19(1)	1(1)	−3(1)	−3(1)
C(37)	14(1)	28(1)	28(1)	−5(1)	−2(1)	5(1)
C(38)	14(1)	17(1)	15(1)	−1(1)	−2(1)	1(1)
C(39)	19(1)	15(1)	18(1)	2(1)	1(1)	3(1)
C(40)	15(1)	20(1)	19(1)	4(1)	−1(1)	−1(1)
O(5)	30(1)	49(1)	60(1)	2(1)	12(1)	0(1)
N(9)	30(1)	42(1)	49(2)	5(1)	13(1)	−2(1)
C(1)	33(2)	40(2)	38(2)	1(1)	9(1)	−7(1)
C(2)	36(2)	66(3)	85(3)	22(2)	16(2)	12(2)
C(3)	67(3)	60(2)	48(2)	−7(2)	15(2)	6(2)
O(6)	23(1)	39(1)	35(1)	2(1)	−10(1)	2(1)
N(1)	23(1)	45(1)	30(1)	−6(1)	4(1)	−4(1)
C(5)	22(1)	68(2)	32(2)	11(1)	−9(1)	0(1)
C(6)	20(1)	37(1)	31(1)	5(1)	3(1)	5(1)
C(11)	47(2)	58(2)	61(2)	−28(2)	20(2)	−18(2)
O(7)	45(1)	54(2)	71(2)	−10(1)	30(1)	4(1)
N(7)	45(2)	34(1)	44(1)	−3(1)	16(1)	−4(1)
C(4)	40(2)	37(2)	49(2)	−3(1)	11(2)	−1(1)
C(7)	110(4)	30(2)	66(2)	−2(2)	43(2)	3(2)
C(8)	107(4)	41(2)	65(2)	7(2)	50(2)	10(2)

$U_{ij} = \exp(-2\pi^2[h^2 (a^*)^2 U_{11} + k^2 (b^*)^2 U_{22} + \ldots + 2hka^*b^* U_{12}])$.

Table S8. Fractional atomic coordinates (×10[4]) and isotropic thermal factors (×10[3]) of hydrogen atoms for compound **1**.

**Atoms**	**X**	**Y**	**Z**	**U_{iso} Å^2**
H(2)	−2808	−1026	−16,020	54
H(10)	541	2741	−8005	23
H(13)	406	−3995	−8879	22
H(16)	396	−2957	−8926	21
H(19)	−1608	−76	−9744	28
H(25)	−1272	−1782	−10,002	20
H(27)	541	1706	−8021	22
H(28)	−1107	536	−11,382	22
H(33)	−1235	−1188	−12,857	22
H(35)	−1877	−1227	−14,494	23
H(36)	−791	−2572	−8780	21
H(37)	−2243	−74	−11,457	29
H(39)	−625	1321	−10,110	22
H(1)	−2431	−2132	−12,221	44
H(2A)	−3104	−1642	−10,614	93
H(2B)	−3006	−1647	−12,350	93
H(2C)	−2977	−1045	−11,351	93
H(3A)	−2071	−1383	−8644	88
H(3B)	−2461	−1556	−8097	88
H(3C)	−2428	−928	−8909	88
H(5A)	−1192	−6340	−5383	65
H(5B)	−1048	−5953	−3786	65
H(5C)	−792	−5974	−5103	65
H(6)	−1733	−5189	−7459	36
H(11A)	−856	−4832	−5426	81
H(11B)	−1123	−4799	−4153	81
H(11C)	−1290	−4570	−5958	81
H(4)	−1234	−3545	−4220	50
H(7A)	−1422	−2287	−2864	97
H(7B)	−1577	−2949	−2834	97
H(7C)	−1853	−2457	−3868	97
H(8A)	−1356	−2437	−7055	99
H(8B)	−1284	−1946	−5671	99
H(8C)	−1721	−2151	−6516	99

$U_{iso} = \exp[-8\pi^2 U(sen\theta/\lambda)^2]$.
